# Characterization of Mechanically Matched Hydrogel Coatings to Improve the Biocompatibility of Neural Implants

**DOI:** 10.1038/s41598-017-02107-2

**Published:** 2017-05-16

**Authors:** Kevin C. Spencer, Jay C. Sy, Khalil B. Ramadi, Ann M. Graybiel, Robert Langer, Michael J. Cima

**Affiliations:** 1 Department of Materials Science and Engineering, Massachusetts Institute of Technology, Cambridge, MA 02139 USA; 2 Koch Institute for Integrative Cancer Research, Massachusetts Institute of Technology, Cambridge, MA 02139 USA; 3 Department of Biomedical Engineering, Rutgers University, Piscataway, NJ Harvard, USA; 4 Harvard-MIT Division of Health Sciences and Technology, Cambridge, MA 02139 USA; 5 McGovern Institute for Brain Research and Department of Brain and Cognitive Sciences, Massachusetts Institute of Technology, Cambridge, MA 02139 USA; 6 Department of Chemical Engineering, Massachusetts Institute of Technology, Cambridge, MA 02139 USA

## Abstract

Glial scar is a significant barrier to neural implant function. Micromotion between the implant and tissue is suspected to be a key driver of glial scar formation around neural implants. This study explores the ability of soft hydrogel coatings to modulate glial scar formation by reducing local strain. PEG hydrogels with controllable thickness and elastic moduli were formed on the surface of neural probes. These coatings significantly reduced the local strain resulting from micromotion around the implants. Coated implants were found to significantly reduce scarring *in vivo*, compared to hard implants of identical diameter. Increasing implant diameter was found to significantly increase scarring for glass implants, as well as increase local BBB permeability, increase macrophage activation, and decrease the local neural density. These results highlight the tradeoff in mechanical benefit with the size effects from increasing the overall diameter following the addition of a hydrogel coating. This study emphasizes the importance of both mechanical and geometric factors of neural implants on chronic timescales.

## Introduction

Neural implants developed in recent years have shown great promise to improve and restore quality of life for millions of patients around the world. These implants have been demonstrated as potential solutions to treat neurological disorders^1^, restore limb function^2^, and unravel the complexities of neural circuits^3^.

These devices can interface with neurons both chemically and electrically and need to operate chronically, especially considering the invasive nature of the implantation process^4^. The implant must operate safely and effectively in the presence of any biological response to the implant for months to years. Many studies have characterized the brain’s immune response to the presence of the neural implant known as astrogliosis, which results in the formation of glial scar directly surrounding the implant^5,6^. The immune response can be divided into two phases – the acute phase and the chronic phase.

The acute response is primarily dominated by microglia and occurs over the first few weeks following implantation. The microglia release pro-inflammatory cytokines and reactive oxygen species (ROS) in response to the implantation injury. These species promote inflammation, negatively affect neuron viability in the vicinity of the implant^5^, and may impact device integrity^7^.

The chronic response is observed at longer implantation times (>4 weeks). The most notable feature of the chronic immune response is the formation and densification of a glial scar around the implant. Reactive astrocytes, characterized by their up-regulation of glial fibrillary acidic protein (GFAP) and the increased production of chondroitin sulfate proteoglycans (CSPG), surround and isolate the injury site from the rest of the neural tissue^5,8,9^. The glial sheath, which is typically a few hundred microns thick, becomes dense around 4–6 weeks and remains stable for the duration of the implantation^10,11^. This process is analogous to the fibrous encapsulation that is observed around implants in other parts of the body^12–14^.

Glial scar forms with the function of protecting the rest of the central nervous system from the foreign body within the tissue as well as from reactive species released following injury to the brain^5,15,16^. Despite these acute benefits, glial scar formation is a key contributor to neural implant failure. Nearly half of all recording implants fail within 6 months of implantation despite initially operating correctly due to glial scar formation^17,18^. Glial scar displaces neurons near the implant. Neurons typically need to be within 100 μm of an electrode to be recorded, although it is generally understood that neurons must be within 50 μm to effectively isolate single unit activity^19,20^. The presence of glial scar decreases the probability that neural signals can be detected by recording electrodes^21^. Glial scar is also known to increase the impedance of the tissue. This effect reduces the volume of tissue activated by electrical stimulation by up to 50% for a typical set of Deep Brain Stimulation (DBS) parameters^22^. A higher current is then required to produce a therapeutic effect on a given neural circuit. These higher charge densities can lead to local tissue damage through electrochemical reactions or physiological changes in response to neural excitation^19,23,24^. Glial scarring is also known to negatively affect the chemical diffusion properties in the tissue surrounding the implant, which could, in turn, negatively affect drug distribution surrounding neural implants. For example, Sykova *et al*. used ion selective electrode measurements to determine that astrogliosis locally increases tortuosity, extracellular space volume fraction, and decreases cellular uptake^8,25,26^.

Conventional neural implant materials have elastic moduli that are many orders of magnitude higher than that of brain tissue (Brain = 5 kPa^27^ vs Tungsten = 400 GPa^28^). Brain tissue is constantly undergoing micromotion (up to 40 μm in magnitude in rats) due to respiration, vascular pulses, and rotational accelerations^29,30^. This persistent relative motion between the implant and tissue is thought to play a major role in directing the chronic response through constant aggravation of local inflammatory cells and damage to local vasculature. Recent work in our group has demonstrated that astrocytes are mechanically responsive to the strain produced from micromotion^31^. Micromotion around neural implants was simulated using high precision linear actuators within a 3D neural culture. Astrocytes directly around the implant were found to undergo hypertrophy (increased area and perimeter) compared to control wells after one week in culture.

The local strain from micromotion results in local mechanical damage that drives scar formation. Finite element analysis (FEA) simulations have been conducted to investigate the effect that mechanical mismatch has on the surrounding tissue^27,32^. These simulations estimate the amount of strain that the brain tissue experiences as a result of brain micromotion in the presence of neural implants with various mechanical properties. Subbaroyan *et al*. conducted simulations that suggested that a probe composed of a hypothetical soft material with modulus of 6 MPa results in a strain two orders of magnitude less than that of a silicon probe (E = 200 GPa)^27^. These results suggest that neural implant designs could incorporate materials with lower mechanical stiffness and/or coatings that promote adhesion with neural tissue to reduce the extent of glial scar formation.

There have been many studies conducted with the goal of reducing the scar response to implanted electrodes with results of varying success, reviewed in depth elsewhere^5,33^. Strategies include local drug delivery^34,35^, using implants that are flexible^36,37^ or mechanically adaptive^38,39^, using degradable shuttles to facilitate implantation of thin implants^40,41^, incorporation of coatings to improve tissue integration^42–45^, modifying surface permeability to inflammatory molecules^46^, and the reduction of implant density^47^. These studies provide insight into the mechanisms of scar formation, and provide suggestions regarding device properties that are important to consider when designing chronic neural implants. There is, however, a clear need for guidance on how key implant design parameters affect the glial scar response around the implant, specifically when considering strategies to modulate the mechanical effects from micromotion.

This study investigates how device dimensions and mechanical properties modulate the immune response in the brain, specifically in the context of using hydrogel coatings to mitigate mechanical damage from micromotion. Thick (25–100 μm) polyethylene glycol dimethacrylate (PEG-DMA) hydrogel coatings, with mechanical properties close to that of brain tissue, were formed on neural implants. PEG based hydrogels were chosen for this study as they are known to be biocompatible in the brain^48,49^ as well as the fact that their properties (chemical, electrical, mechanical) may be controlled based on formulation^50–52^. The mechanical properties of these coatings was characterized to confirm that the elastic modulus was matched to that of brain tissue. The capacity to reduce local strain fields was investigated *in vitro*, and the acute and chronic *in vivo* response to these coatings was investigated in a rodent cranial implantation model. Previous studies have only explored polymer coatings at a certain dimension^53,54^, therefore comparing implants with both differing diameters and mechanical properties. To that end, this study incorporates size-based controls to decouple the size and mechanical effects of 10–100 μm scale coatings. Together, these experiments seek to elucidate a clear framework of geometric and mechanical parameters for optimal device performance.

## Materials and Methods

### Materials

Polyethylene glycol (MW 2000, 4000, 8000), methacrylic anhydride, triethyl amine, 3-(trichlorosilyl) propyl methacrylate, 2-Hydroxy-4′-(2-hydroxyethoxy)-2-methylpropiophenone, agarose, and all solvents used in this study were purchased from Sigma Aldrich (St. Louis, MO, USA) and were ACS grade. All chemicals and antibodies were used as-received unless noted otherwise. 150 μm and 400 μm OD glass capillaries were purchased from Vitrocom, Inc (Mountain Lakes, NJ, USA). 200 μm capillaries were purchased from Hampton Research (Aliso Viejo, CA, USA). All glass capillaries were sterilized via autoclave upon receiving from manufacturer prior to any handling.

### Synthesis of PEG-Dimethacrylate

Polyethylene glycol dimethacrylate (PEG-DMA) was synthesized according to a procedure adapted from Lin-Gibson, *et al*.^55^. Briefly, 10 g of PEG (MW 700–8000) was dissolved in approximately 30 ml of anhydrous dichloromethane. The solution was reacted with 2.2 molar equivalents of methacrylic anhydride and triethyl amine (0.4 ml) over activated molecular sieves (3 g). The reaction was allowed to proceed to completion under nitrogen atmosphere (4 days at room temperature). The solution was filtered via vacuum filtration, and approximately 20 ml of dichloromethane was removed via rotary evaporation. The PEG-DMA was precipitated in ice- cold diethyl ether. The product was dried under vacuum at room temperature overnight to remove residual solvent prior to subsequent use.

### Formation of PEG Hydrogel on Glass Capillaries

**Figure 1:**
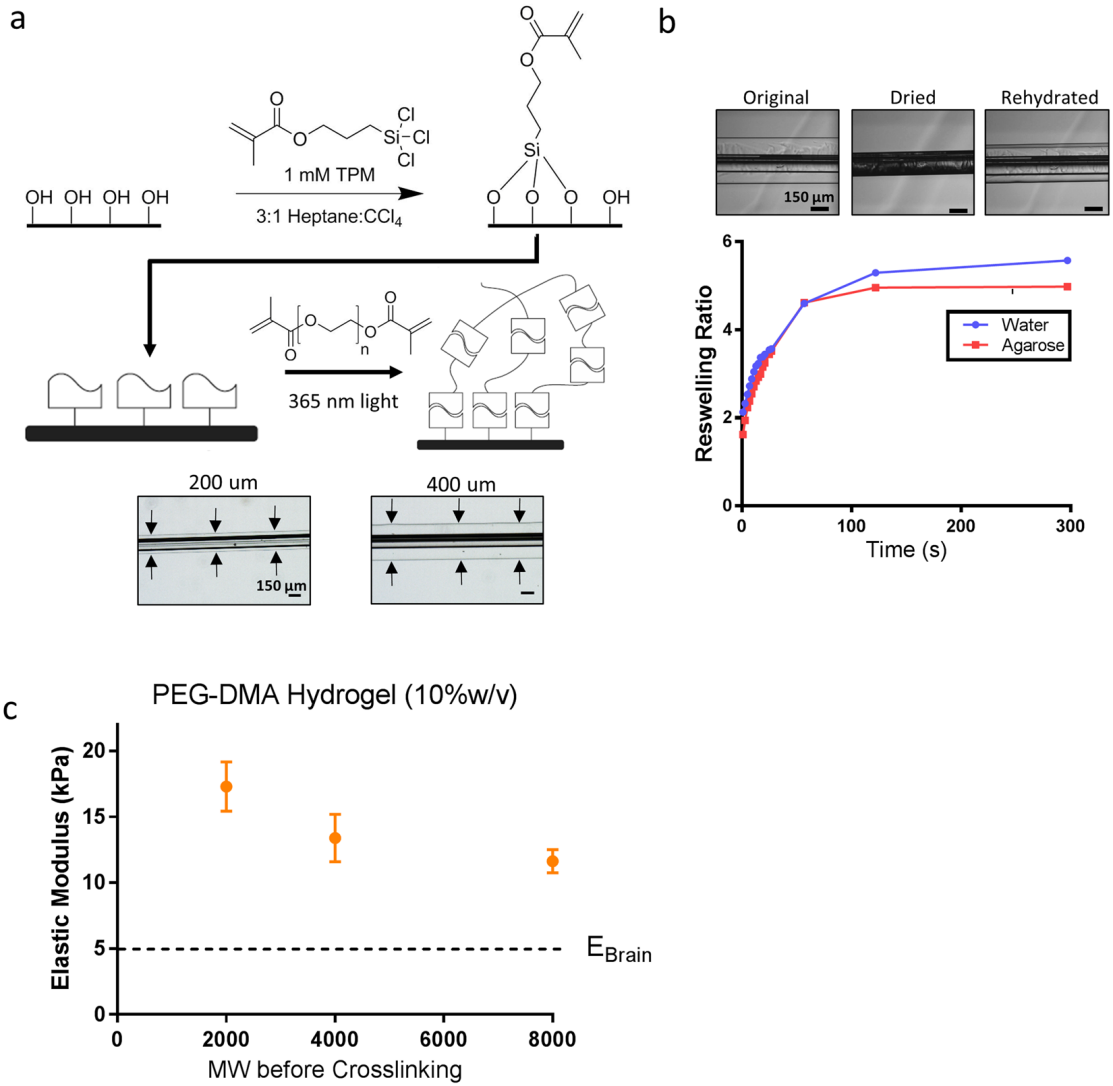
Hydrogel Formation Procedure and Mechanical Characterization. (**a**) Soft PEG hydrogel coatings were formed on borosilicate glass capillaries through a multistep process. The surface of the glass is piranha etched followed by treatment with 1 mM TPM. An aqueous PEG dimethacrylate solution is then filled into a cylindrical mold containing the treated capillaries. Exposure to UV light crosslinks the polymer network and forms the hydrogel on the surface of the device. The thickness of the hydrogels can be readily controlled by adjusting mold geometry. Representative bright field images of the hydrogel images are shown below the reaction scheme (scale bar = 150 μm) (**b**) The hydrogel coatings are dehydrated prior to implantation to maintain coating integrity. The kinetics of rehydration was monitored in water and a 0.6% agarose brain tissue phantom at room temperature (scale bar = 150 μm) (**c**) The elastic modulus of the hydrogel was measured via Hertzian analysis of AFM force curves at several PEG-DMA chain lengths. The elastic modulus of the coatings is controlled by adjusting the polymer concentration before crosslinking and the PEG-DMA molecular weight. 10% PEG-DMA hydrogels (MW 8000) were used for subsequent *in vivo* experiments (E = 11.6 kPa).

PEG-DMA hydrogel coatings were formed on the surface of the glass capillaries (Fig. 1a).

Glass capillaries were etched in piranha solution (3:1 ratio of concentrated sulfuric acid to 30% hydrogen peroxide) for five minutes. The capillaries were then washed three times in DI water, and dried under nitrogen atmosphere.

The cleaned glass capillaries were treated with 1 mM 3-(trichlorosilyl) propyl methacrylate (TPM) in a 4:1 ratio of heptane-carbon tetrachloride under N_2_ atmosphere for 10 minutes. The capillaries were then washed in hexane, acetone, and water. The TPM treatment covalently functionalized the glass surface with methacrylate functional handles to improve hydrogel adhesion. The TPM-functionalized glass capillaries were stored under inert gas prior to hydrogel formation.

Hydrogel precursor solution was prepared by combining PEG-DMA in water (5–20% weight/volume) with the photo initiator 2-Hydroxy-4′-(2-hydroxyethoxy)-2-methylpropiophenone (0.5% w/v). Treated capillaries were placed in a cylindrical glass mold (200–400 μm I.D.). The mold was filled with hydrogel precursor solution. The solution was exposed to a UV light source (Cure Spot 50, Dymax Corp, Torington, CT, USA) for 60 s to crosslink the polymer network and form the hydrogels on the glass device. The coated capillaries were removed from the mold, and were stored in PBS until experimental use. The main defect observed throughout the gel formation process were shearing of the coating during removal of these molds, as well as the glass device not being centered in the mold. Both of these defects were easily identified through visual inspection and were not encountered in subsequent experiments.

Devices were cut to approximately 5 mm in length and were polished to have a blunt tip, as they were determined to be stiff enough for tissue penetration without the need for beveling. The devices were attached to micromachined Delrin caps for easy handling, and were sterilized by soaking in 70% ethanol for 24 hours, followed by UV exposure (λ = 254 nm) for one hour prior to implantation. Devices were washed three times in sterile tissue culture grade water, and hydrogel coatings were dehydrated by exposure to air prior to *in vivo* implantation studies. Gels were dehydrated in ambient air for 24 hours, much longer than the time necessary for visual dehydration (≈15 min). Air temperature or humidity was not controlled beyond standard building HVAC conditions.

### Mechanical Characterization of PEG Hydrogels

The Young’s modulus of the PEG-DMA hydrogels was measured via analysis of atomic force microscopy (AFM) force curves. Planar hydrogels (approximately 100 μm thick) were formed on TPM treated borosilicate glass slides for use in the AFM measurements. Hydrogels were loaded into the AFM (Veeco, Nanoscope IV with picoforce scanner head, Oyster Bay, NY), and the tip (k = 14 N/m with functionalized 45 μm bead polystyrene tip, Novascan) was brought into contact with the sample surface. The tip deflection was measured as a function of indentation depth over the course of a 1.5 μm z-displacement. The elastic modulus was then determined via Hertzian analysis of the force-displacement curve^56^.

### *In Vitro* Strain Field Measurements

A custom micromotion simulation device was used to examine the strain reduction capabilities of the hydrogel coatings^57^. A 0.6% agarose brain phantom with embedded polystyrene beads (0.05% w/v, 6 μm, Polysciences Inc, Warrington, PA) was formed around uncoated and hydrogel coated devices, which were mechanically linked to a linear actuator. Agarose phantoms at this concentration are known to have mechanical properties similar to brain tissue^58^. The devices were displaced 30 μm at a frequency of 2 Hz in the perpendicular and axial directions to simulate micromotion due to rotational accelerations and respiration/vascular pulsations respectively^29,30^. A series of bright field images were obtained before and after displacement. The images were analyzed via the Particle Image Velocimetry (PIV) plugin for ImageJ to construct the displacement fields around the device. Strain fields were measured for uncoated devices (GC150), 200 μm hydrogel coated devices (P10–200), and 400 μm hydrogel coated devices (P10–400) in both directions of motion.

### Device Implantation in Rodent Brain

All animal studies were conducted in accordance with the guidelines of the Committee on Animal Care at Massachusetts Institute of Technology, as well as the NIH Guide for the Care and Use of Laboratory Animals. All experimental protocols were reviewed and approved by the Committee on Animal Care at Massachusetts Institute of Technology before the initiation of experiments.

All instruments used in the surgical procedures were autoclaved and sterilized between animals using a glass bead heat sterilizer. Two devices were implanted (one per hemisphere) for each animal used in this study.

Adult female Fisher F344 rats (150–175 g, Charles River Laboratory, Wilmington, MA) were anesthetized via isoflurane (2–3% mixed with oxygen). The animal’s head was shaved and disinfected with alternating scrubs of betadine and isopropanol (3× each). The animal was transferred to a stereotactic frame. An incision was made along the midline to expose the skull. A high-speed drill was used to perform the two craniotomies at coordinates of 2 mm posterior, 2 mm lateral to Bregma. The dura mater was removed using a bent 31 gauge needle. Bleeding was stopped before device implantation, and the brain surface was irrigated with sterile saline. The devices were lowered into the brain (approximately 0.5 mm/second) until the plastic handling cap was in contact with the skull, a depth of approximately 5 mm. Excessive bleeding during and following implantation was not commonly observed as hemostasis was generally achieved less than 2 min after implantation. Three animals were excluded due to excessive blood being observed around the implant site following device retrieval out of a total of approximately 60 rats. The devices were fixed to the skull using Metabond and Cement-It dental cement (Pentron, Orange, CA). Care was taken to ensure that the cement did not have sharp edues or rough areas that may have irritated the surrounding area. The skin was pulled over the dried cement and sealed with 5–0 monofilament nylon sutures. Buprenorphine-SR (1.0 mg/kg) was provided to the animal as an analgesic for 72 hours post implantation. No meloxicam was administered to the animals in this study to avoid the NSAID from affecting the inflammatory processes.

The naming convention used in this study for the hydrogel coated samples is P(Concentration)-Swollen Thickness. For example, P10–200 refers to a device coated with a 10% PEG hydrogel, with a total thickness of device plus hydrogel of 200 μm. All hydrogel coatings were formed on 150 micron diameter glass capillaries. Samples that start with GC refer to unmodified glass capillaries followed by the diameter in microns (i.e. GC150 = 150 micron, uncoated glass capillary).

Experimental groups for the animal studies consisted of glass capillaries with diameters ranging between 150 μm, 200 μm, and 400 μm (GC150, GC200, GC400) and PEG-DMA 10% hydrogel coated capillaries with a total thickness of 200 μm and 400 μm (P10–200, P10–400). An initial pilot study of non-coated and P10–200 samples was used to estimate the sample sizes required for statistical power (n = 6). n ≥ 6 for each experimental group and time point unless otherwise noted. The sample size for each cohort is indicated in the figure legend (Figs 3, 4, 5 and 6).

### Animal Euthanasia and Tissue Harvesting

Animals were euthanatized at set time points following device implantation (1 week, 4 weeks, 8 weeks) via CO_2_ asphyxiation. Animals were perfused with approximately 50 ml of PBS followed by 100 ml of 4% paraformaldehyde (PFA) in PBS. The brains were allowed to post-fix in 4% PFA for 48 hours after perfusion prior to removing the devices from the tissue. The brains were placed in sucrose sinking solutions of increasing concentration (10%, 20%, 30% sucrose in PBS) until sinking was observed. Brains were frozen in optimal cutting temperature embedding media by immersion in liquid nitrogen chilled 2-methyl butane. 20 μm thick sections, cut perpendicular to the device axis, were obtained with a cryostat (Leica Microsystems, Buffalo Grove, IL, USA). All sections used examined in this study were obtained at a depth of 0.5–2.0 mm below the surface of the brain, corresponding to the sensory cortex region of the brain^59^. Tissue sections were stored at −80 °C prior to immunostaining.

### Immunohistochemistry

Tissue sections were stained for glial scar/ inflammation markers including glial fibrillary acidic protein (GFAP, 1:100 mouse anti-GFAPx488 Alexaflour, EMD Millipore, Billerica, MA, USA) to assess the extent of scarring, ED1/CD68 (1:300, mouse anti-rat CD68, clone ED1, EMD Millipore, Billerica, MA, USA) to assess activated macrophages, IgG (1:250, Donkey Anti-Rat IgGx647 Alexafluor, Abcam, Cambridge, MA, USA) to assess blood-brain barrier (BBB) permeability, and local neural nuclei NeuN (1:300, rabbit anti-NeuN, EMD Millipore, Billerica, MA, USA). Frozen sections were equilibrated to room temperature, and rehydrated for 10 minutes in PBS-0.5% tween. Sections were incubated in PBS with 5% donkey serum for 1 hour at room temperature to prevent non-specific staining. Primary antibodies were diluted in antibody incubation buffer (PBS containing 1% bovine serum albumin, 1% normal donkey serum, 0.3% triton x-100, and 0.01% sodium azide). Sections were incubated in the primary antibody overnight at 4 °C. Sections were washed three times, and incubated with secondary antibody diluted in incubation buffer (1:300 Donkey Anti-Rabbit x Dylight 650, Abcam, 1:300 Donkey-AntiMousex488 Alexafluor, Millipore) for 1 hour at room temperature. Slides were washed three times and counterstained with Hoechst 33258 (2 μg/ml, Sigma Aldrich, St. Louis, MO, USA) for 15 minutes. Slides were rinsed in PBS and coverslips were mounted with Prolong Gold (Invitrogen, Carlsbad, CA, USA). All stained slides were imaged within 1 week of completing the staining procedure, and were stored away from light at 4 °C to prevent any significant photobleaching.

### Imaging and Data Analysis

Fluorescent stained sections were imaged on the EVOS Fl automated microscope (Thermo Fisher Scientific, Waltham, MA, USA). The device location was identified and stitched images (approximately 4 mm × 4 mm regions) were obtained in the region surrounding the implant site. Exposure settings were chosen to avoid saturation and were maintained for each individual marker. Images were not altered in any way prior to image analysis. The IF images displayed in Figs 3, 4 and 5 are displayed at equal brightness and contrast settings, and have been cropped to 750 × 750 μm regions around the device region to improve visibility.

The staining intensity as a function of distance from the tissue-implant interface was quantified using the MINUTE program, provided by the Capadona lab at Case Western Reserve University (CWRU, Cleveland, OH, USA)^60^. Briefly, the implant interface was defined by the user, and the fluorescent intensity was quantified in 2 μm rings extending from the implant. The total fluorescent intensity was normalized to a region far from the implant site to account for differences in section thickness/staining efficiency. The normalization factor for each section was taken to be the average value between 900–1200 μm away from the implant. This distance was chosen based on initial inspection of the marker intensity profiles and is in line with previously reported studies^60^. Four sections were imaged for each animal to produce an average intensity profile, which was used for subsequent statistical analysis. The area under the curve was segmented into 50 μm binned intervals around the device location.

No randomization or blinding was conducted in this study. The use of the automated MINUTE analysis program helped to ensure that user bias was minimized throughout the analysis process. No data exclusion criteria were applied. The only animals excluded from analysis were animals in which severe surgical trauma had occurred (e.g. excessive blood observed under implant, (n = 3), or major tissue damage occurred during device retrieval (n = 2).

### Statistical Analysis

Observed experimental differences were assessed for statistical significance across all experimental cohorts through a two-way ANOVA test using Prism 6 (In Stat Graphpad, La Jolla, CA, USA). Post hoc analysis (Tukey’s multiple pairwise comparisons test) was performed to compare marker expression between groups at different distances from the device-tissue interface. The Shapiro-Wilk test was conducted to confirm data were normal distributions. Statistical significance was considered p < 0.05. All data presented represents mean ± standard error of the mean unless otherwise indicated.

### Data Availability

All relevant data to this study are available from the corresponding author on reasonable request.

## Results

### Hydrogel Formation and Characterization

PEG-DMA hydrogel coatings were formed on 150 μm borosilicate capillaries via a UV polymerization process (Fig. 1a). The thickness of the coating was controlled by changing the mold geometry. Coatings were produced with a total final diameter of 200 μm and 400 μm, and had minimal variation in thickness along the length of the device (Fig. 1a). It was determined that coatings could be dehydrated prior to implantation to maintain coating integrity. The kinetics of coating rehydration was measured in water and an agarose brain tissue phantom (Fig. 1b). The coating returned to its original dimensions in approximately two minutes, with no significant difference in thickness or morphology. No major differences in the kinetics of swelling were observed between water and agarose tissue phantom.

The elastic modulus of the hydrogels was measured via Hertzian analysis of AFM force curves. The hydrogel formulations tested in this study spanned three different polymer concentrations before crosslinking (5%, 10%, and 20% w/v%) and three different PEG-DMA molecular weights (MW = 2000, 4000, and 8000). These hydrogels had elastic moduli ranging from 1.6 kPa to 171.5 kPa.

Trends observed in the modulus data demonstrate that the elastic modulus may easily be controlled based on reaction conditions. Increasing the polymer concentration before crosslinking increases the resulting modulus of the hydrogel (Supplementary Figure S1). A major increase (approximately 10×) in the modulus was observed between 10% and 20% polymer concentration. A slight decrease in elastic modulus was observed when producing hydrogels with longer length PEG-DMA molecules (Fig. 1c). For subsequent *in vivo* experiments, 10%, MW8000 hydrogels were used which were found to have an elastic modulus of 11.64+/−2.0 kPa.

### *In Vitro* Strain Reduction

**Figure 2:**
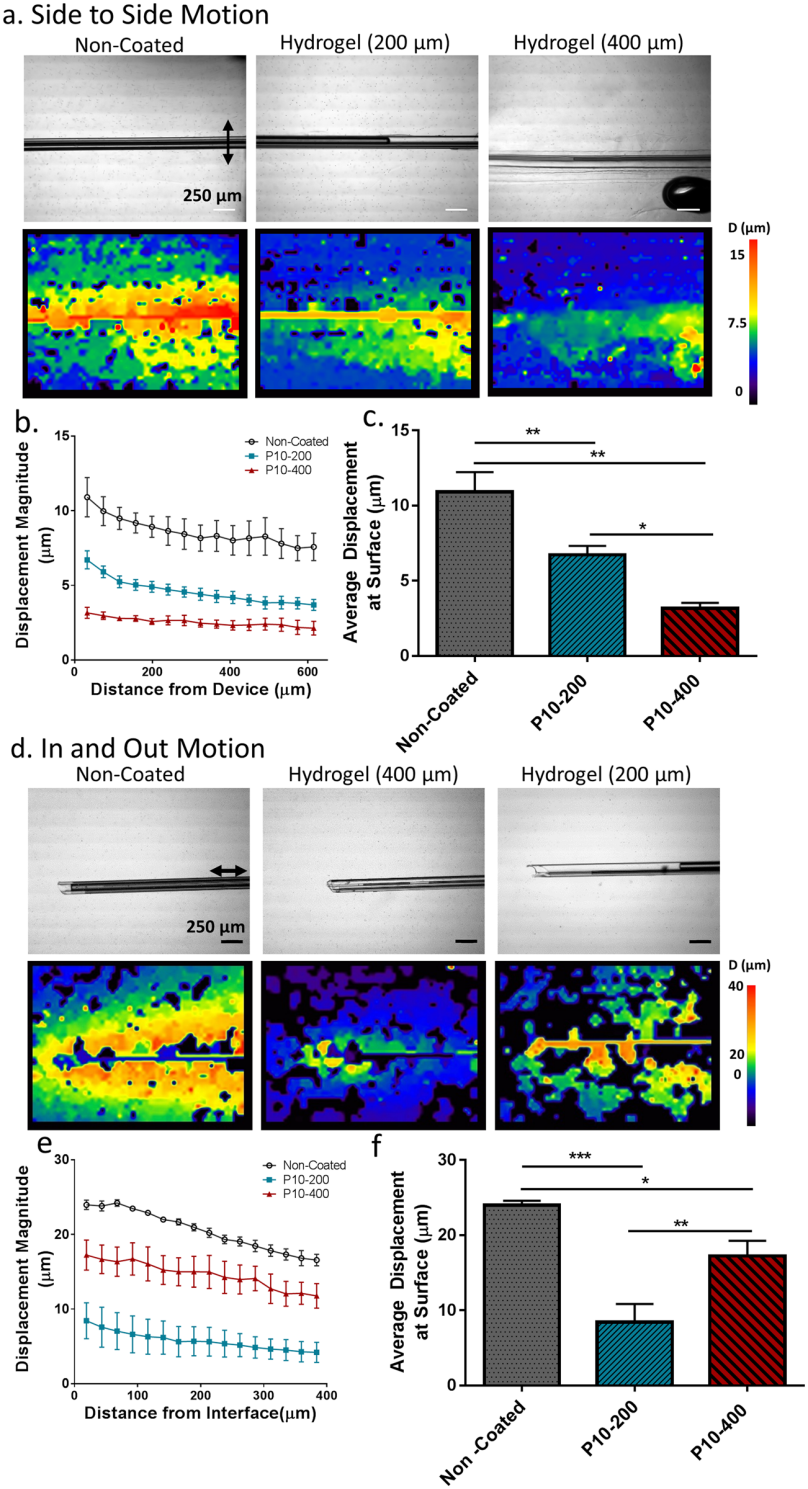
Hydrogel *In Vitro* Characterization. Hydrogel coated devices reduce local strain fields which result from micromotion compared to non-coated controls. Particle image velocimetry was used to quantify the displacement surrounding the device. (**a**) Bright field images and strain field plot overlays following a 30 micron displacement which was applied to devices perpendicular to the device axis embedded within an agarose tissue phantom to mimic micromotion from rotational accelerations. (scale bar 250 μm). (**b**) Average line profiles of the displacement magnitude as a function of distance from the device and (**c**) the average displacement at the surface were calculated. Hydrogel coated samples significantly reduced strain fields around the device. Thicker hydrogel coatings (P10–400) had greater strain reduction compared to thinner hydrogel coatings (P10–200). Sample sizes: non-coated n = 3, P10–400 hydrogel: n = 3, P10–200 μm hydrogel: n = 5. (**d**–**f**) Strain field maps, line profiles, and surface displacement for non-coated and hydrogel coated samples following a 30 μm displacement along the device axis to simulate micromotion from respiration. Both thicknesses of hydrogel coatings significantly reduced the strain fields around the device. Thinner hydrogel coating (P10–200) had greater strain reduction compared to the thicker coating (P10–400) in this direction of motion. Sample sizes non-coated: Samples sizes: Non-coated: n = 4, P10–400: n = 3, P10–200: n = 4. A series of six images was analyzed for each individual experiment. *p < 0.05, **p < 0.01, ***p < 0.001.

The capacity of hydrogel coated devices to reduce the local strain fields that result from micromotion was measured using a custom built *in vitro* micromotion simulation set up^57^. Devices coated with PEG-hydrogels of two thicknesses (200 μm and 400 μm) were coupled to a high precision linear actuator, embedded in a 0.6% agarose brain tissue phantom, and 30 μm displacements were applied along and perpendicular to the device axis. A series of images were taken throughout the motion process. Images at the two extremes of motion were analyzed via the ImageJ PIV plugin to construct the displacement fields around the device (Fig. 2a,d). A clear reduction in the local strain field around hydrogel coated devices was observed for both hydrogel thicknesses in terms of both size and magnitude for both directions of motion. The average displacement at the surface of the device for the non-coated control was 10.9 ± 2.3 μm, 6.7 ± 1.4 μm for the P10–200 group, and 3.2 ± 0.64 μm for the P10–400 group for the side to side motion direction (Fig. 2b). The thicker hydrogel was observed to have reduced strain for all distances from the device investigated in this study.

A slightly different behavior was observed when the devices were displaced in the axial direction. The P10–200 hydrogel samples produced less strain than the P10–400 and non-coated samples (Fig. 2e). The average displacement at the surface for the non-coated control was 24.0 ± 1.2 μm, and was 17.25 ± 3.5 μm for the 400 μm hydrogel sample, and was 8.4 ± 4.8 μm for the 200 μm hydrogel sample (mean ± S.D., Fig. 2d). There was a significant reduction (p < 0.05) between NC and P10–400 up to 190 μm away from the device-tissue interface. The difference in average displacement between the P10–200 hydrogel sample and the non-coated sample was found to be statistically significant (p < 0.01) for all distances investigated in this study.

### *In Vivo* Animal Study Results

#### Size Control Hydrogel Study

**Figure 3:**
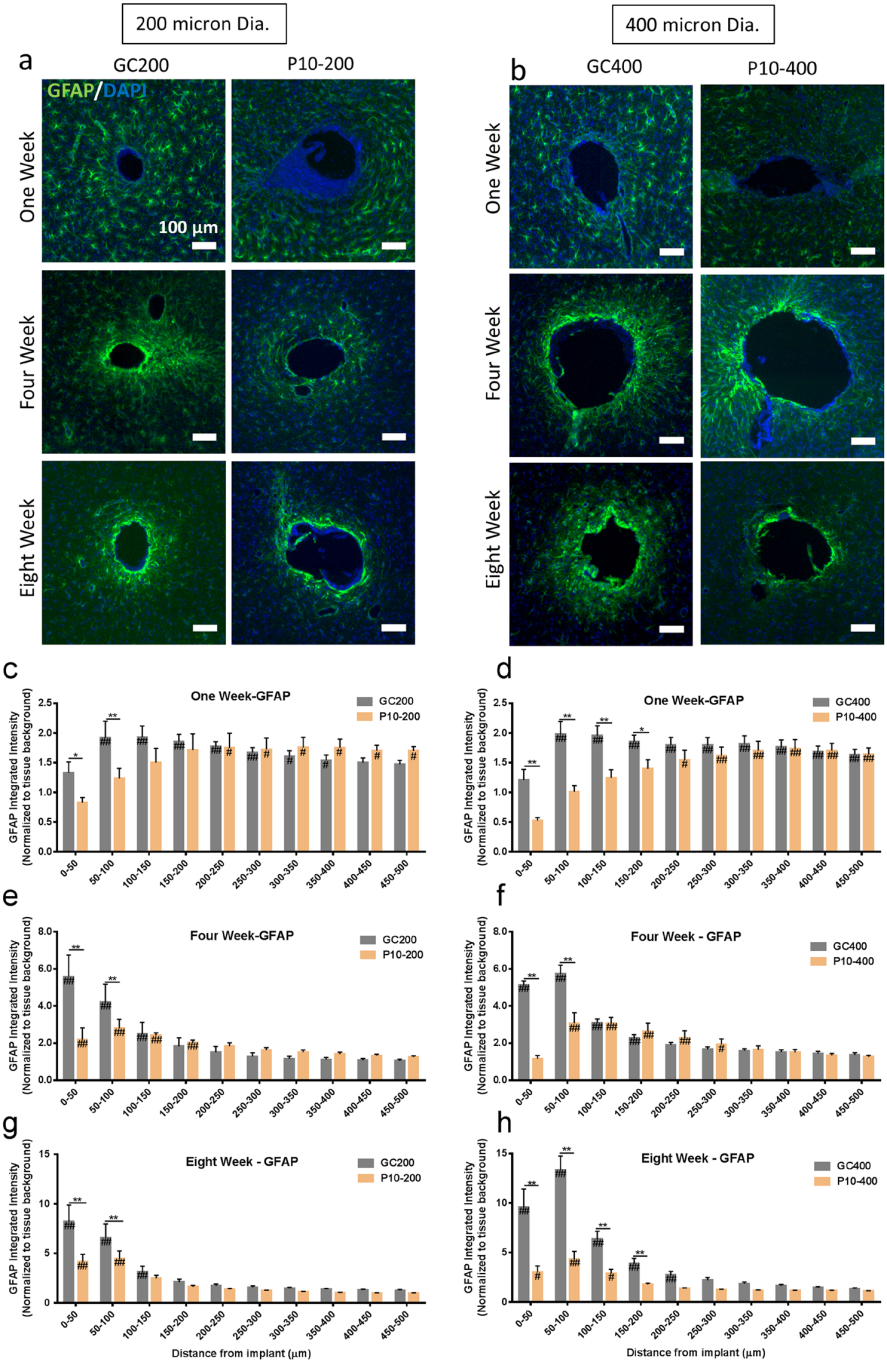
Effect of Implant Modulus on Scarring Soft implants produce less glial scarring compared to hard implants when diameter is held constant. GFAP expression was quantified as a function of distance from the device tissue interface for implants for PEG hydrogel coated implants (E ≈ 10 kPa) and glass capillaries (E ≈ 70 kPa) of identical diameter (d = 200 um and d = 400 um) at 1, 4, 8 weeks post implantation. (**a**,**b**) Representative immunofluorescence images depicting the GFAP reactivity at the implant location at 1, 4, and 8 weeks post implantation. Scale bar = 100 μm. Glial scarring was significantly reduced for 200 μm and 400 μm hydrogel coated implants at 1, 4, and 8 weeks post implantation. (**c**,**d**) The average GFAP reactivity as a function of distance from the device at 1 week post implantation. (**e**,**f**) The average GFAP reactivity as a function of distance from the device at 4 weeks post implantation. (**g**,**h**) The average GFAP reactivity at eight weeks post implantation. **p < 0.01, *p < 0.05 indicates a statistically significant difference between cohorts at a given distance from the implant interface. ^##^p < 0.01, ^#^p < 0.05 indicates a statistically significant difference compared to background. Cohort sizes (1 wk, 4 wk, 8 wk), GC200 = (6, 5, 7) P10–200 = (6, 7, 7), GC400 = (6, 5, 10), P10–400 = (6, 6, 6). Each n corresponds to an individual animal. A minimum of four sections were analyzed per animal.

**Figure 4:**
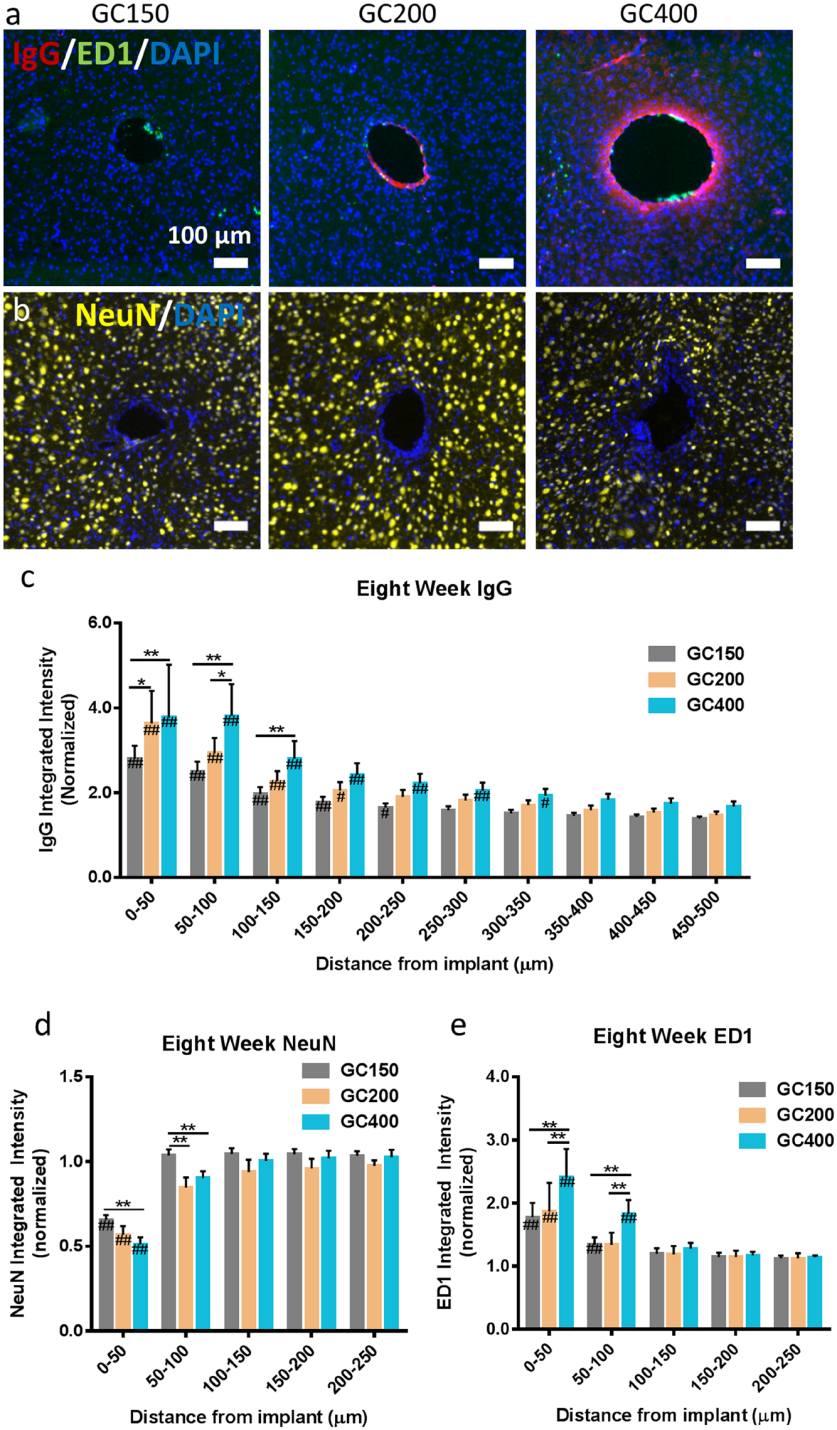
Implant Diameter Comparison. Increasing implant diameter increases scarring at 8 weeks post implantation when implant modulus is held constant. Glass capillaries with diameters ranging from 150 μm to 400 μm were implanted into the rodent brain and analyzed at 1, 4, and 8 weeks post implantation. (**a**) The GFAP activity as a function of distance from the implant at 1 week. A slight decrease in the GFAP reactivity was observed directly around the implants for the larger capillaries. (**b**) GFAP reactivity at four weeks post implantation. GFAP reactivity was increased around the implant for 400 μm implants at 4 weeks post implantation. The GC400 group had increased scarring compared to GC200 and GC150 groups in the 100 μm surrounding the implant. (**c**) Representative IF images (scale bar (100 μm) of the GFAP reactivity at 8 weeks post implantation. (**d**) GFAP reactivity at 8 weeks post implantation. Increased diameter (200 μm and 400 μm) implants were found to produce increased scarring at eight weeks post implantation. **p < 0.01, *p < 0.05 indicates a statistically significant difference between cohorts at a given distance from the implant interface. ^##^p < 0.01, ^#^p < 0.05 indicates a statistically significant difference compared to background. Cohort sizes (1 wk, 4 wk, 8 wk). GC150 = (11, 11, 18), GC200 = (6, 5, 7), GC400 = (6, 5, 10). Each n corresponds to an individual animal (biological replicate). A minimum of four sections were analyzed per animal.

**Figure 5:**
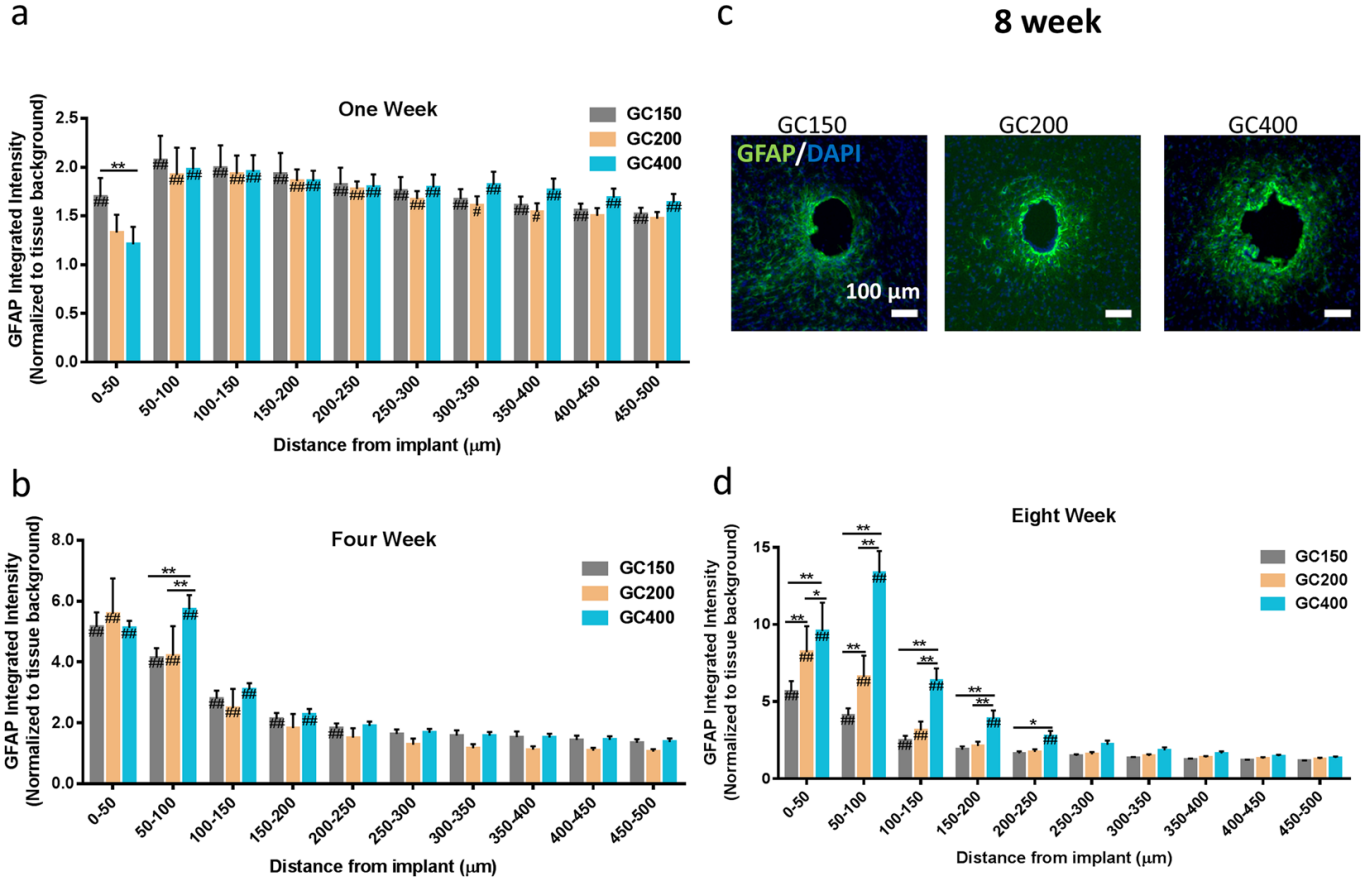
Increasing implant diameter has additional effects on the biological response at 8 weeks post implantation. (**a**) Representative IF images of the IgG and ED1 reactivity around implants at 8 weeks post implantation (scale bar 100 μm). (**b**) Representative IF images of the NeuN staining around glass capillary implants at 8 weeks post implantation. (Scale bar 100 μm). (**c**,**d**,**e**) IgG, NeuN, and ED1 staining around the implants at eight weeks post implantation. Animals with increased diameter implants were found to have increased BBB permeability, IgG (**d**) decreased neural density, and (**e**) increased activated macrophages staining in the region directly surrounding the implant location. **p < 0.01, *p < 0.05 indicates a statistically significant difference between cohorts at a given distance from the implant interface. No significant differences were observed beyond the distances shown in the figures. ^##^p < 0.01, ^#^p < 0.05 indicates a statistically significant difference compared to background. Cohort sizes: GC150: n = 18, GC200: n = 7, GC400: n = 10. Each n corresponds to an individual animal. A minimum of four sections were analyzed per animal.

**Figure 6:**
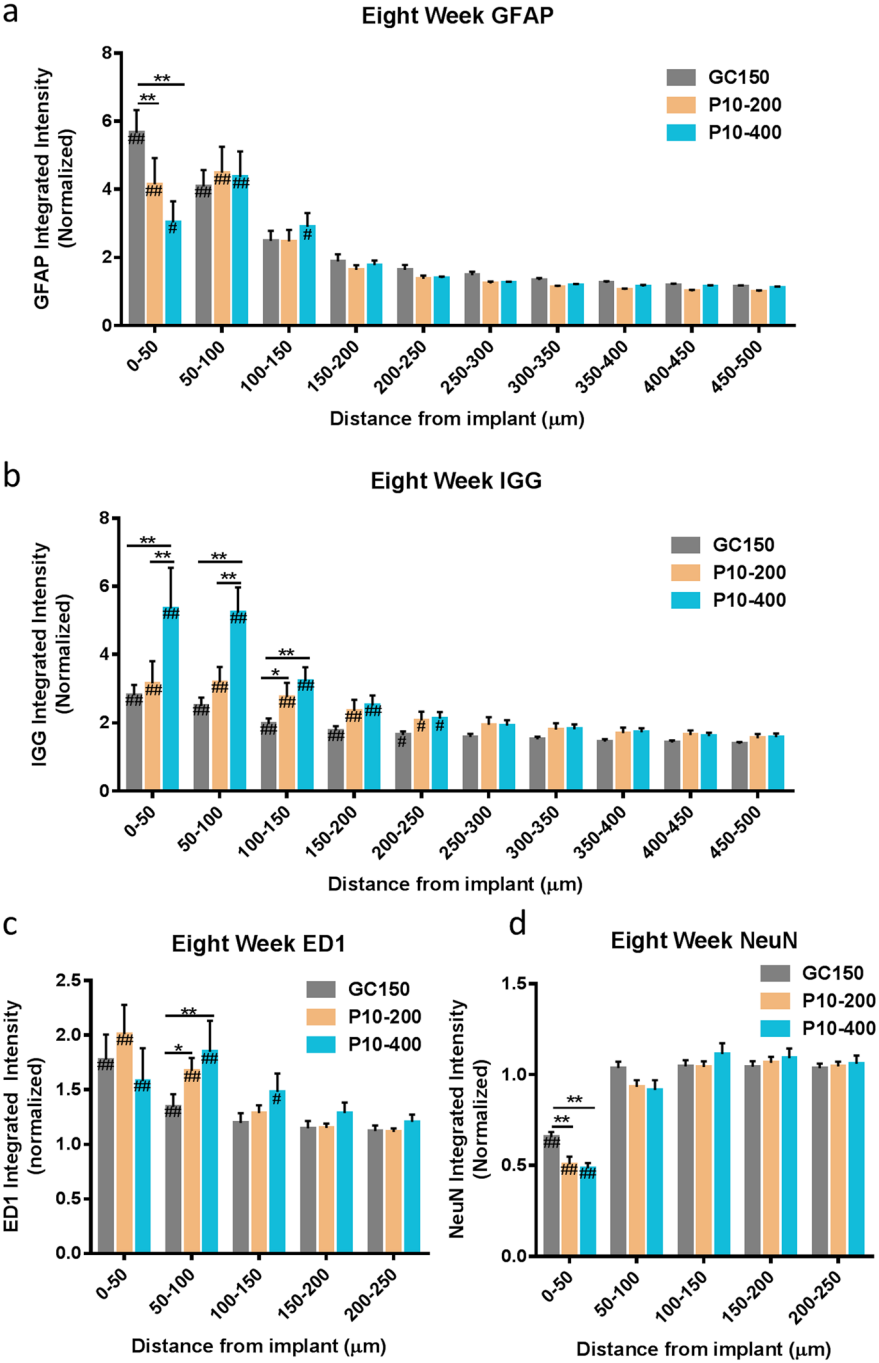
The response to thick hydrogel coatings on smaller diameter implants exhibits both mechanical and size effects at eight weeks post implantation. (**a**) Mechanically matched hydrogel coatings reduce GFAP at 8 weeks post implantation compared to smaller diameter hard implants. Effects from the increased implant size of the hydrogel coatings were also observed. The hydrogel coated implants had (**b**) increased IgG staining directly surrounding the implant, (**c**) increased macrophage activity, and (**d**) decreased neural density compared to the 150 micron diameter glass capillaries. These results highlight the tradeoff in mechanical benefits and size effects from increasing the overall implant diameter following the addition of a hydrogel coating. **p < 0.01, *p < 0.05 indicates a statistically significant difference between cohorts at a given distance from the implant interface. ^##^p < 0.01, ^#^p < 0.05 indicates a statistically significant difference compared to background. No significant differences were observed beyond the distances shown in the figures. Cohort sizes GC150: n = 18, P10–200: n = 7, P10–400: n = 6. Each n corresponds to an individual animal. A minimum of four sections were analyzed per animal.

The first comparison made in the *in vivo* studies was to assess the impact that implant modulus has on scar formation when the implant diameter is held constant. Figure 3 shows representative IHC images of the tissue reaction observed at 1, 4, and 8 weeks post implantation for glass capillaries (GC200, GC400) and hydrogel coated samples (P10–200, P10–400) (Fig. 3a,b). The MINUTE program (provided by the Capadona Lab at CWRU) was used to quantify amount of GFAP staining as a function of distance from the device-tissue interface (Fig. 3c–h). The marker intensity profiles displayed (Figs 3, 4, 5 and 6) were truncated to focus on regions surrounding the implant in which statistically significant differences were observed between experimental groups. All intensity profiles were confirmed to return to baseline expression levels beyond the displayed regions in the graphs.

Both hydrogel coated samples (P10–200 and P10–400) samples showed reduced GFAP reactivity at 1, 4, and 8 weeks post implantation when compared to the glass capillary implants (GC200 and GC400). P10–200 was significantly reduced compared to GC200 up to 100 μm from the implant at 1, 4, 8 weeks post implantation (Fig. 3c,e,g). The scarring in the P10–200 samples was reduced by 50% at four weeks post implantation, and 40% at eight weeks compared to the GC200 samples. P10–400 had significantly reduced scarred compared to GC400 up to 200 microns away from the implant at one week post implantation (Fig. 3d). GFAP staining for the P10–400 samples at four weeks post implantation was significantly reduced (approximately 60%) in the 100 microns surrounding the implant interface (Fig. 3f). P10–400 samples at eight weeks post implantation were found to have a 60% decrease in GFAP staining up to 200 microns away from the implant (Fig. 3h).

### Glass Capillary Size Study

#### GFAP

Glass capillaries of three different sizes (GC150, GC200, and GC400) were implanted into the rodent brain for 1, 4, and 8 weeks in order to investigate the effect that implant diameter has on the glial scar reaction, when implant modulus is held constant. Figure 4c shows representative IF images of the GFAP reactivity around the three different implants at 8 weeks post implantation. The size of the implant did not have a major impact on GFAP staining at one week post implantation (Fig. 4a). The GC400 had a 30% significant reduction in GFAP intensity compared to the GC150 sample in the 50 microns surrounding the implant. No other significant differences were observed at one week post implantation. The GC400 at four weeks post implantation had a slightly larger tissue reaction compared to GC150, with a 1.4 fold increase in GFAP intensity being observed 50–100 microns away from the implant (Fig. 4b). A clear size effect in the scarring reaction was observed at eight weeks post implantation (Fig. 4d). GC200 had increased GFAP staining compared to GC150 up to 100 microns away (1.5 fold increase) from the implant-tissue interface. The 400 micron capillary showed the greatest scarring reaction, with statistically significant increase over GC150 up to 250 microns away from the implant. The GC400 samples exhibited a 2.3 fold increase in GFAP intensity compared to GC150 samples over this distance. GC400 had approximately 1.5 fold higher GFAP levels compared to GC200 in the 100 microns surrounding the implant.

#### Additional Size Effects

The effect that size has on other inflammatory markers including IgG, ED1/CD68, and NeuN at eight weeks post implantation was also measured. Tissue sections were stained for IgG as an indicator for BBB permeability. Tissue sections were stained for IgG and ED1 (Fig. 5a) as well as NeuN (Fig. 5b). All three diameter implants showed some evidence of elevated BBB permeability with IgG > 2.5 × of background levels in regions directly surrounding the implant (Fig. 5c). IgG values were statistically significant compared to background up to 200 μm away from the implant for all diameters. Larger implants were found to have greater BBB permeability compared to the smaller diameter implants. The GC400 implant had greater a 1.5 fold increase in IgG staining up for 150 microns from the tissue interface compared to the GC150 group. The GC200 group had a 1.3 fold increase in IgG staining in the 50 microns directly surrounding the implant interface (p < 0.05) compared to the GC150 group. Larger implants showed a slight, but statistically significant decrease in the neural nuclei density in the 100 microns surrounding the implant (Fig. 5d). In the immediate vicinity of the implant (0–50 microns away), GC150 had NeuN staining which was 65.6% of background, GC200 had NeuN staining of 56% of background, and GC400 was 51.2% of background. Implant diameter also had an effect on the presence of activated macrophages around the device-tissue interface. The GC400 group had a 1.4 fold increase ED1 staining in the 100 microns around the implant compared to the GC150 group (Fig. 5e). No statistically significant difference was observed between the GC200 and GC150 implants.

### Hydrogel Coating Study

The final comparison made in this study was between GC150 capillaries and hydrogel coated samples (Fig. 6). Both the P10–200 and P10–400 hydrogel sample at eight weeks post implantation were found to have reduced GFAP immunoreactivity in the 50 microns directly surrounding the implant (Fig. 6a). The P10–200 group exhibited a 22% reduction compared to the GC150 capillaries. The P10–400 group was found to have a 46% reduction compared to the GC150 capillaries. There was no significant difference in regions farther from the implant between any of the three groups. The larger hydrogel coated implants showed elevated levels of IgG in the tissue surrounding the implant (Fig. 6b). The P10–400 group had a 1.9 fold increase in IgG staining compared to the GC150 group in the 150 μm surrounding the implants. The P10–200 group showed a 40% increase in IgG staining in 100–150 μm from the implant interface. Hydrogel coated samples also had a larger region with activated macrophages compared to the non-coated GC150 sample (Fig. 6c). There was no statistical difference between the three groups immediately surrounding the implant, whereas both hydrogel coated samples had elevated ED1 staining 50–100 microns away from the interface (P < 0.05). The P10–200 group had a 25% increase and the P10–400 group had a 40% increase compared to the GC150 group at this distance from the implant. The hydrogel-coated samples had a statistically significant reduction in neural cell density in the 50 microns directly surrounding the implant (Fig. 6d). The NeuN staining was 50% of background levels for the P10–200 group, and 48% of background for the P10–400 group, compared to 65% for the GC150 group. No differences in NeuN staining were observed farther away from the device tissue interface.

## Discussion

The PEG-DMA hydrogels formed in this experiment have controllable mechanical properties that are on the order of brain tissue^27^, and many orders of magnitude less than those of conventional neural implants. The tunability of the elastic properties allowed the hydrogel properties to be optimized, whether it be matching the modulus to a certain region of the brain^61^, or to match the properties of the local tissue at a certain critical time post implantation^62^. The elastic modulus measurements and trends observed in our studies are similar to those that have been previously reported in literature^55^. There is a significant increase in the mechanical properties as the polymer concentration increases (Supplementary Figure S1), due to an increase in the number of physical crosslinks in the material. The modulus decreases with increasing PEG-DMA molecular weight, for a given polymer concentration (Fig. 1c). This effect is less pronounced compared to adjusting the polymer concentration, suggesting that physical crosslinks play a major factor in the molecular structure of this hydrogel. The large increase between 10% and 20% w/v before crosslinking, is suggestive of a transition in hydrogel microstructure which has been previously described in literature^52^.

An essential characteristic of the PEG-DMA hydrogel coating system is the capacity of the coating to be dehydrated prior to implantation, preventing damage to the gel from the shear forces produced during implantation. The two-minute time scale for swelling (Fig. 1b) was sufficient time to implant the device in the region of interest under stereotaxic guidance. No damage was observed to coated implants when they were removed from the brain at the end of the experiment. The swelling observed *in vivo* may be slower than observed in the agarose gel as the free water content in brain tissue is less than the agarose tissue phantom. The time to complete swelling could be increased through addition of a soluble coating on top of the hydrogel layer. The use of a silane-methacrylate surface treatment enables the gel to be covalently bonded to the surface of the implant, helping to ensure integrity of the coating throughout the duration of implantation. Both coating thicknesses investigated in this study were observed to be intact throughout the duration of implantation. No damage to the hydrogel coatings was observed upon retrieval at all timepoints.

The average device diameter measured in histological sections matched the approximate diameter of the hydrogel coatings suggesting that minimal damage or degradation had occurred during the implantation process and throughout the 8 week implantation period investigated in this study (Supplementary Figure S2). The variation observed in individual histological sections (Figs 3, 4 and 5) is thought to be due to variations in the tissue processing and sectioning process. The holes observed in the GC implant samples often observed to be slightly smaller than that of the diameter matched hydrogel. This phenomenon could be due to the presence of residual tension arising from astrocytes and other cells pulling on the hard glass surface, causing the tissue to slightly retract once the device has been removed. This effect would not occur as significantly at the soft gel interface, leading to their hole diameters being more in line with the nominal implanted dimensions. The use of the MINUTE program, which quantifies the reactivity in terms of distance from the device-tissue interface, was used to minimize the effects of tissue void size on the analysis procedure.

The coating process used in this study yields coatings that are conformal and have minimal variation in thickness. A similar approach could be used to coat metal implants^63^, silicon chips^64^, or polymer implants^65,66^ by slightly modifying the chemistry. The cylindrical symmetry of the devices used in this experiment lent itself well to the cast molding process. More complex geometries could be coated with PEG-DMA hydrogels through a dip coating, or spray coating process (Supplementary Figures S3).

A 400 micron coating thickness was used based on estimations using PEG hydrogel mechanical properties. PEG hydrogels can undergo at least 25% strain^67^ within the elastic deformation regime, thus in order to fully absorb 30 micron displacements (typical maximum displacements observed by Gilleti *et al*.^30^), the hydrogel coating should be at least 120 microns thick according to the following equation:

1 $$target\,thickness=\frac{{d}_{micromotion}}{{\varepsilon }_{Max}}=\frac{30\,\mu m}{0.25}=120\,\mu m$$

In ongoing work, we are using150 micron capillaries for brain probes, yielding ~400 micron diameter implants (2*125 micron thick coatings + 150 micron glass).

The *in vitro* strain model enabled the degree of strain reduction to be quantified for a given set of micromotion parameters (Fig. 2). The use of an agarose brain phantom and high precision linear actuator enables the user to simulate the local effects of micromotion that are difficult to observe *in vivo*. Previous strategies to estimate micromotion effects include simulation studies^27^, as well force measurements conducted *in vivo* ^68^. An *in vitro* approach, such as the one developed in this experiment, has advantages, as it enables different engineering parameters related to device geometry and mechanical properties to be rapidly optimized without the need for large scale animal studies. The results of the strain field study clearly show that the reducing the effective elastic modulus through the addition of a thick hydrogel coating significantly reduces the local strain around the device. It is logical that these diminished strains should result in less glial reactivity and scarring around the implant based on the documented mechanical responsiveness of astrocytes^69^. Both hydrogel thicknesses had reduced strain compared to controls in both axial and perpendicular displacement. Differing behavior was observed between the two modes of motion. The thicker P10–400 group had greater reduced strain compared to the P10–200 group when the devices were displaced perpendicular to their axis. The increased coating thickness provided an increased mechanical buffer to reduce the strain from the micromotion. The 200 μm hydrogel produced less strain than the 400 μm hydrogel coating around the implant when the implants were displaced in the axial direction. This functional result highlights the role of friction and shear between the implant and tissue when the device is displaced in the axial direction. Axial micromotion predominantly arises *in vivo* from respiration and vascular pulsation, while perpendicular displacements occur as the result of rotational accelerations^29,30^. These results show that it is important to consider both modes of displacement when incorporating features to mitigate the effects of micromotion *in vivo*.

The *in vivo* animal data presented in this study identify several key factors that one should consider when designing neural implants to minimize scarring around neural probes. Softer implants with a lower elastic modulus produce less scarring at 1, 4, and 8 weeks post implantation (Fig. 3) at both diameters investigated (200 micron and 400 micron). This effect has been observed in literature before in studies demonstrating that mechanically adaptive devices produce less scarring at 8 weeks post implantation^70^. Softer implants reduce scarring due to the decrease in strain around the implant, supported by our *in vitro* data and previously conducted studies^30,68^. Less mechanical aggravation of the glial cells is occurring directly surrounding the implant. The relative difference between the hard and soft implants was greater in case of the 400 micron implants. The GC 400 produced 3.13x more scarring within 100 μm of the implant compared to the P10–400 implants, while the GC200 samples produced 1.7x more scarring than the P10–200 implants at 8 weeks post implantation. This effect is likely due in part to a greater relative difference in strain fields between the hydrogel coated and glass implants as the implant footprint increases. There are other potential contributing factors, in addition to purely mechanical effects, which may also be a factor in the performance of the hydrogel coatings in this study. These include the ability of the hydrogel to buffer the concentration of local inflammatory molecules around the implant^46^ and the reduced density of the hydrogel compared to the glass capillary^47^. All of these factors are likely play a role in directing the inflammatory response and should be considered in future probe designs.

The glass capillary results reported here show the significance of neural probe diameter with regard to the chronic glial response. Larger implants have increased scarring at 8 weeks post implantation (Fig. 4), a finding that is consistent with previously published studies. Thelin *et al*. found that larger diameter tungsten electrodes had GFAP scarring at 6 and 12 weeks post implantation, as well as increased ED1 and decreased NeuN staining^71^. Our findings show previously unrecognized effect of implant duration; the effect that size has on glial scarring becomes more pronounced at longer time points. The initial injury is comparable between the three groups on this size scale, but the response at longer time points is dominated by differences in local strain.

Larger implants also produced greater numbers of activated macrophages and increased BBB permeability (Fig. 5). Previous studies have suggested that soluble factors released from activated macrophages may drive changes in local BBB integrity^72^. The increased presence of activated macrophages may also negatively affect the implant over time (11, 65). The additional inflammation for larger implants also leads to a decrease in the amount of viable neurons around the implant, which could reduce the effectiveness of the implant.

The tradeoff in mechanical benefits and strain reduction must be balanced with size effects from increasing the implant footprint when considering using materials systems as a coating (Fig. 6). A thick, hydrogel coating reduces scarring around the implant at 8 weeks post implantation. This will provide benefits to implant function including a reduction in local impedance, an increase in drug diffusivity, and an improvement in the recording capabilities of the implant. Size effects similar to those documented in the glass capillary study, such as increased BBB permeability and increased activated macrophage presence, were observed in the larger hydrogel coatings groups. The reduction in the number of neurons adjacent to the device could also impact the ability to successfully modulate neural activity both locally and on the circuit level. Whether the decrease in neural cell density leads to a clinically significant decrease in device function should be further investigated within the framework of the intended application.

The slight reduction in neural nuclei density should certainly be considered in recording applications, as this may have negative impacts on the ability to record and isolate single unit activity. The presence of the coating itself may push viable neurons away from the recording site of the probe as PEG hydrogels aren’t naturally conductive^50^. This obstacle may be potentially overcome through incorporation of conducting polymers into the coating itself ^51,73^. Additionally, many neural implants make recording measurements primarily from the tip of the device, but the neural density at the just beyond the end of the device is a neglected factor that should be considered. A reduction in scarring around the length of the device could improve recording capabilities near the tip of the device; reactive cells along the length of the device contribute to the overall inflammatory response through release of proinflammatory cytokines and ROS.

The primary focus of this study was to investigate the mechanical and size effects of hydrogel coatings. The aqueous nature of the PEG-hydrogel formation process could, however, be adapted to encapsulate therapeutic molecules to further improve the biological response to implants. Cells^74^, proteins^75^, adhesion molecules^76^, small molecule drugs^77^ could either be linked to the hydrogel surface, or encapsulated for controlled release to further improve the biological response to the neural implant. Drug delivery strategies could be targeted to reduce macrophage activation^78,79^, astrocyte reactivity^80^, or BBB permeability^81^. Flexible implants^37^, engineering features to reduce local strain^36^, or mechanically adaptive composites^70^ could be alternative strategies to capture some mechanical benefits without significantly altering the dimensions of the implant. The *in vitro* strain model discussed here would be a good tool to compare the strain reduction capabilities of each of these approaches.

The overall objective of this study was to characterize the benefits of both modulus and size in reducing the scar reaction around neural implants. The data obtained in this study indicates that benefits can be achieved by reducing the elastic modulus of the device (Fig. 3) or the overall dimensions of the device (Fig. 4). While soft and small are seemingly ideal characteristics of a neural probes, other considerations must also be taken into account given the intended application for the probe. Implants which are too small in diameter may be too flexible to accurately target deep brain structures, thus requiring novel techniques for implantation. Rigid devices, which may be accurately implanted, would benefit from incorporation of soft hydrogel coatings to serve as a mechanical buffer between the probe and tissue. Large, electrically inert soft coatings may result in the isolation of electrodes from the targeted neural cell bodies despite reducing the extent of scar formation. This issue may be overcome through the development of novel probe designs which incorporate flexible electrical leads within the coating itself. The benefits and drawbacks of using mechanically matched coatings that significantly alter the dimensions of the implant approach should be carefully considered depending on the specific application.

## Conclusion

This study reports on the effect of implant modulus and diameter on the chronic reaction to neural implants by to coating conventionally hard borosilicate implants with PEG-DMA hydrogels with an elastic modulus close to that of brain tissue. Reducing the elastic modulus of neural implants leads to less scarring at chronic time points by minimizing the effects of micromotion induced strain fields around the implant. When considering coatings that significantly alter the final dimensions of the implant, there is a tradeoff between the mechanical benefits of strain reduction and the increased diameter of the coating. Taken together, these results highlight the importance of both reducing the dimensions of implants as well as incorporating novel materials to reduce mechanical damage from micromotion around the implant.

## Electronic supplementary material

Supplementary FigureSet
