# Erratum: Characterization of Mechanically Matched Hydrogel Coatings to Improve the Biocompatibility of Neural Implants

**DOI:** 10.1038/s41598-017-12312-8

**Published:** 2017-10-09

**Authors:** Kevin C. Spencer, Jay C. Sy, Khalil B. Ramadi, Ann M. Graybiel, Robert Langer, Michael J. Cima

**Affiliations:** 1 Department of Materials Science and Engineering, Massachusetts Institute of Technology, Cambridge, MA 02139 USA; 2 Koch Institute for Integrative Cancer Research, Massachusetts Institute of Technology, Cambridge, MA 02139 USA; 3 Department of Biomedical Engineering, Rutgers University, Piscataway, NJ Harvard, USA; 4 Harvard-MIT Division of Health Sciences and Technology, Cambridge, MA 02139 USA; 5 McGovern Institute for Brain Research and Department of Brain and Cognitive Sciences, Massachusetts Institute of Technology, Cambridge, MA 02139 USA; 6 Department of Chemical Engineering, Massachusetts Institute of Technology, Cambridge, MA 02139 USA

*Scientific Reports* **7**:1952; doi:10.1038/s41598-017-02107-2; Article published online 16 May 2017

This Article contains an error in the order of the Figures 4 and 5. Figures 4 and 5 were published as Figures 5 and 4 respectively. The correct Figures appear below as Figures 1 and 2. The Figure legends are correct.

**Figure 1:**
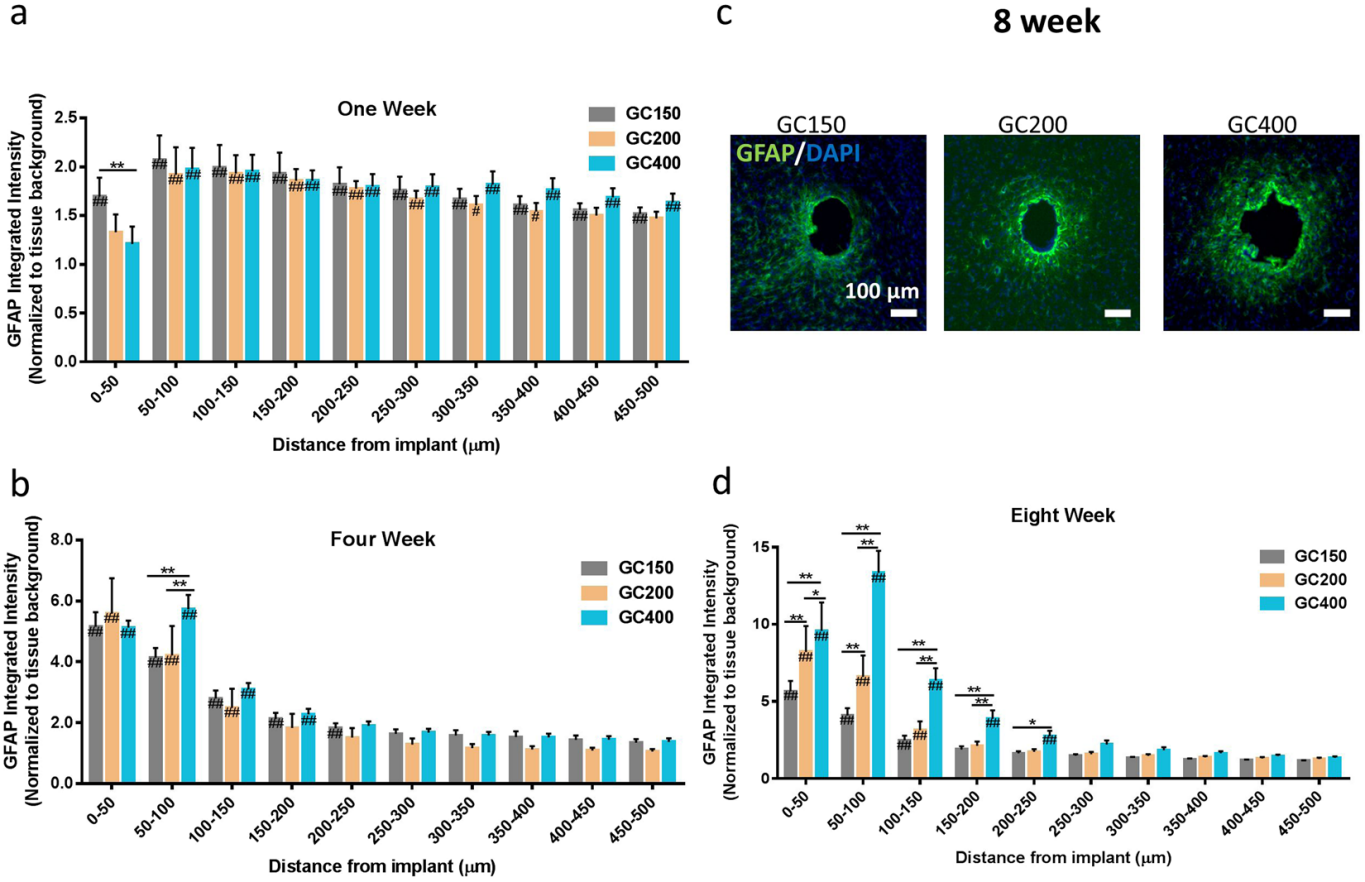
Implant Diameter Comparison. Increasing implant diameter increases scarring at 8 weeks post implantation when implant modulus is held constant. Glass capillaries with diameters ranging from 150 μm to 400 μm were implanted into the rodent brain and analyzed at 1, 4, and 8 weeks post implantation. (**a**) The GFAP activity as a function of distance from the implant at 1 week. A slight decrease in the GFAP reactivity was observed directly around the implants for the larger capillaries. (**b**) GFAP reactivity at four weeks post implantation. GFAP reactivity was increased around the implant for 400 μm implants at 4 weeks post implantation. The GC400 group had increased scarring compared to GC200 and GC150 groups in the 100 μm surrounding the implant. (**c**) Representative IF images (scale bar (100 μm) of the GFAP reactivity at 8 weeks post implantation. (**d**) GFAP reactivity at 8 weeks post implantation. Increased diameter (200 μm and 400 μm) implants were found to produce increased scarring at eight weeks post implantation. **p < 0.01, *p < 0.05 indicates a statistically significant difference between cohorts at a given distance from the implant interface. ^##^p < 0.01, ^#^p < 0.05 indicates a statistically significant difference compared to background. Cohort sizes (1 wk, 4 wk, 8 wk). GC150 = (11, 11, 18), GC200 = (6, 5, 7), GC400 = (6, 5, 10). Each n corresponds to an individual animal (biological replicate). A minimum of four sections were analyzed per animal.

**Figure 2:**
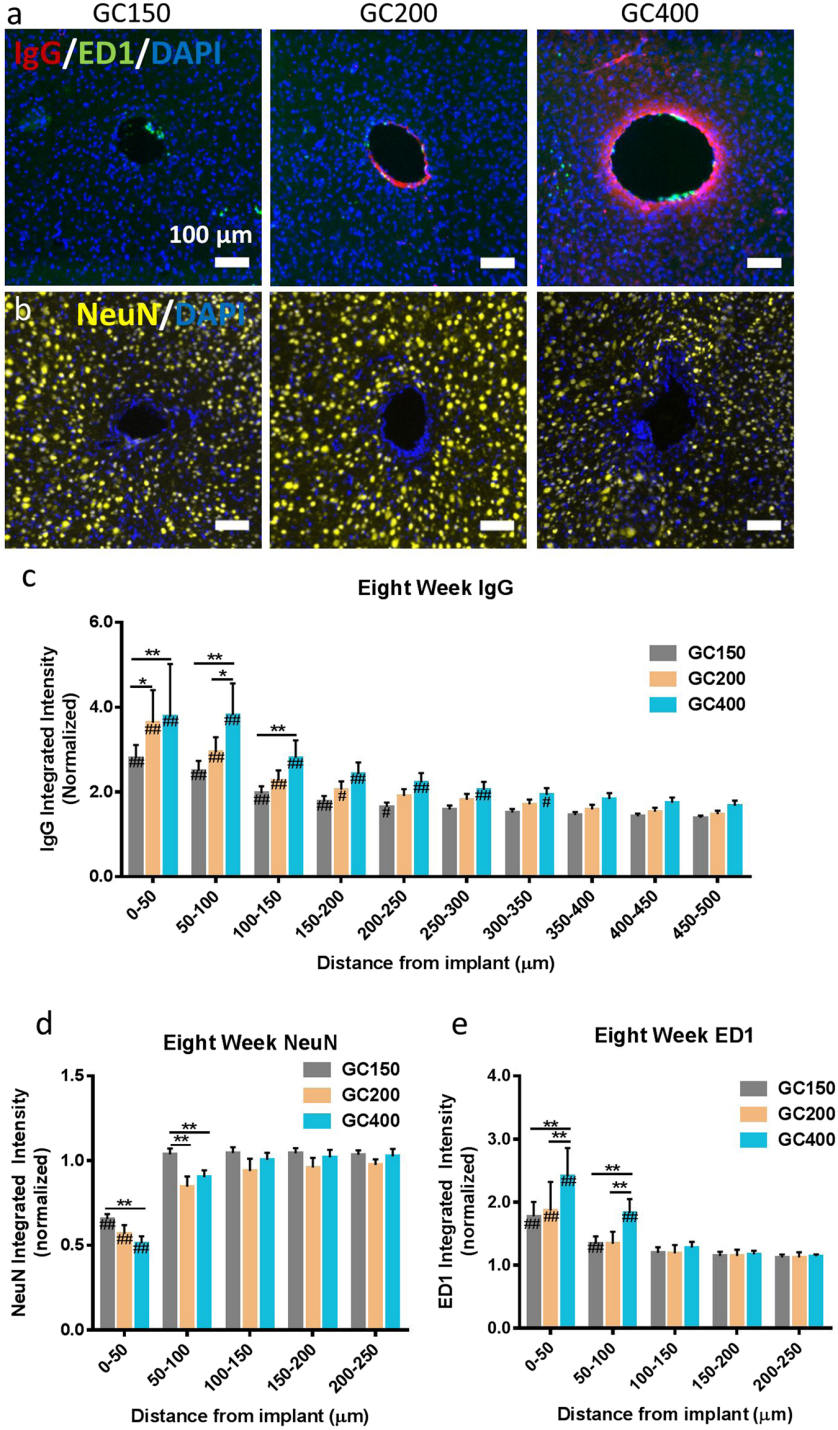
Increasing implant diameter has additional effects on the biological response at 8 weeks post implantation. (**a**) Representative IF images of the IgG and ED1 reactivity around implants at 8 weeks post implantation (scale bar 100 μm). (**b**) Representative IF images of the NeuN staining around glass capillary implants at 8 weeks post implantation. (Scale bar 100 μm). (**c**,**d**,**e**) IgG, NeuN, and ED1 staining around the implants at eight weeks post implantation. Animals with increased diameter implants were found to have increased BBB permeability, IgG (**d**) decreased neural density, and (**e**) increased activated macrophages staining in the region directly surrounding the implant location. **p < 0.01, *p < 0.05 indicates a statistically significant difference between cohorts at a given distance from the implant interface. No significant differences were observed beyond the distances shown in the figures. ^##^p < 0.01, ^#^p < 0.05 indicates a statistically significant difference compared to background. Cohort sizes: GC150: n = 18, GC200: n = 7, GC400: n = 10. Each n corresponds to an individual animal. A minimum of four sections were analyzed per animal.

