# Supplementary material for: Characterization of Mechanically Matched Hydrogel Coatings to Improve the Biocompatibility of Neural Implants
Source: Sci Rep. 2017 May 16;7:1952. doi: 10.1038/s41598-017-02107-2 (PMC5434064; doi:10.1038/s41598-017-02107-2)
Supplement: Supplementary file 1 — Supplementary FigureSet [file 41598_2017_2107_MOESM1_ESM.pdf]

Characterization of Mechanically Matched Hydrogel Coatings to Improve the Biocompatibility of Neural Implants

Kevin C. Spencer[a], Jay C. Sy[b,c] Khalil Rhamadi[d], Ann M. Graybiel[e], Robert Langer[b,f], Michael J. Cima[*,b,a]

a. Department of Materials Science and Engineering, Massachusetts Institute of Technology, Cambridge, MA 02139
b. Koch Institute for Integrative Cancer Research, Massachusetts Institute of Technology, Cambridge, MA 02139.
c. Department of Biomedical Engineering, Rutgers University, Piscataway, NJ Harvard–
d. MIT Health Sciences and Technology Division, Cambridge, MA 02139, USA.
e. McGovern Institute for Brain Research and Department of Brain and Cognitive Sciences, Massachusetts Institute of Technology, Cambridge, MA 02139.
f. Department of Chemical Engineering, Massachusetts Institute of Technology, Cambridge, MA 02139.

* To whom correspondence should be addressed. mjcima@mit.edu

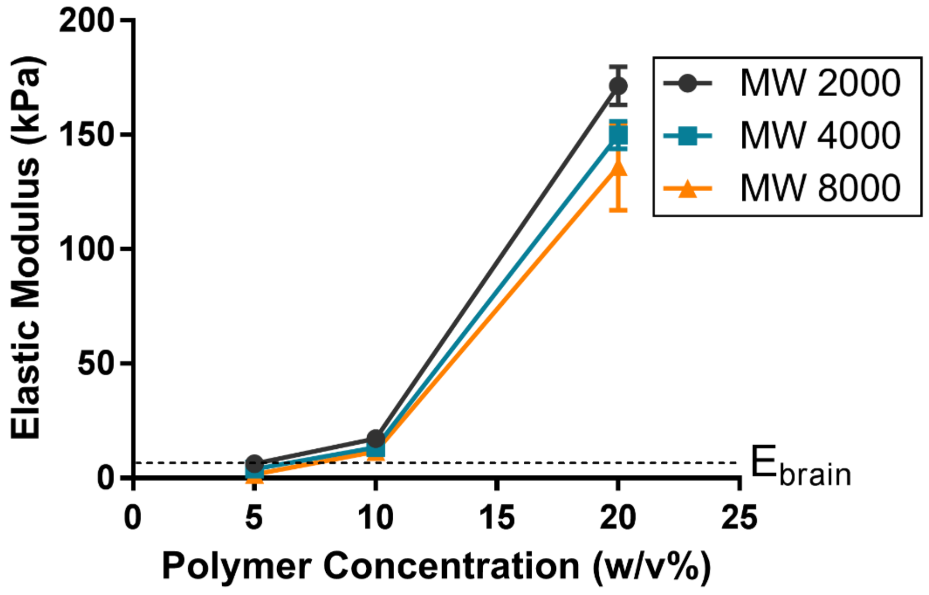

Supplementary Figure S1: The elastic modulus of various PEG-DMA hydrogel formulations was measured by analyzing AFM force curves. The elastic modulus of the coatings is controlled by adjusting the polymer concentration before crosslinking and the PEG-DMA molecular weight. A significant increase in the elastic modulus was observed between 10% and 20% w/v. A PEG-DMA hydrogel with MW8000 10% hydrogel was chosen for *in vivo* studies. Error bars mean +/- S.E.M.

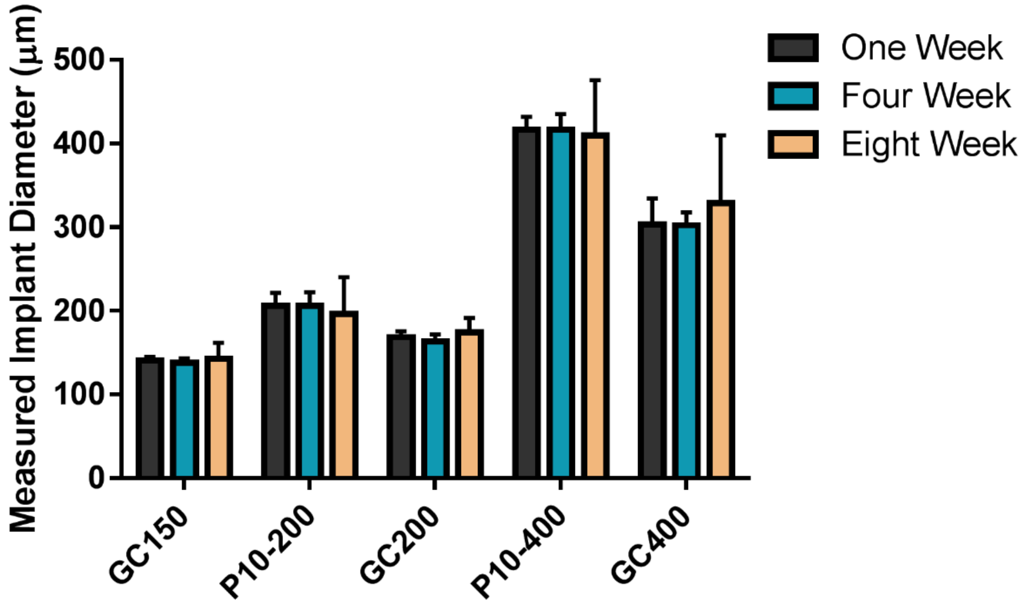

Supplementary Figure S2: Measured Implant Diameter for glass capillary and hydrogel coated samples at one, four, and eight weeks post implantation. These values are the size of the device location within the histological sections. The values were measured via imageJ. No significant degradation in coating integrity was observed over the course of implantation for the hydrogel coated samples. Error bars indicate the standard deviation of the mean.

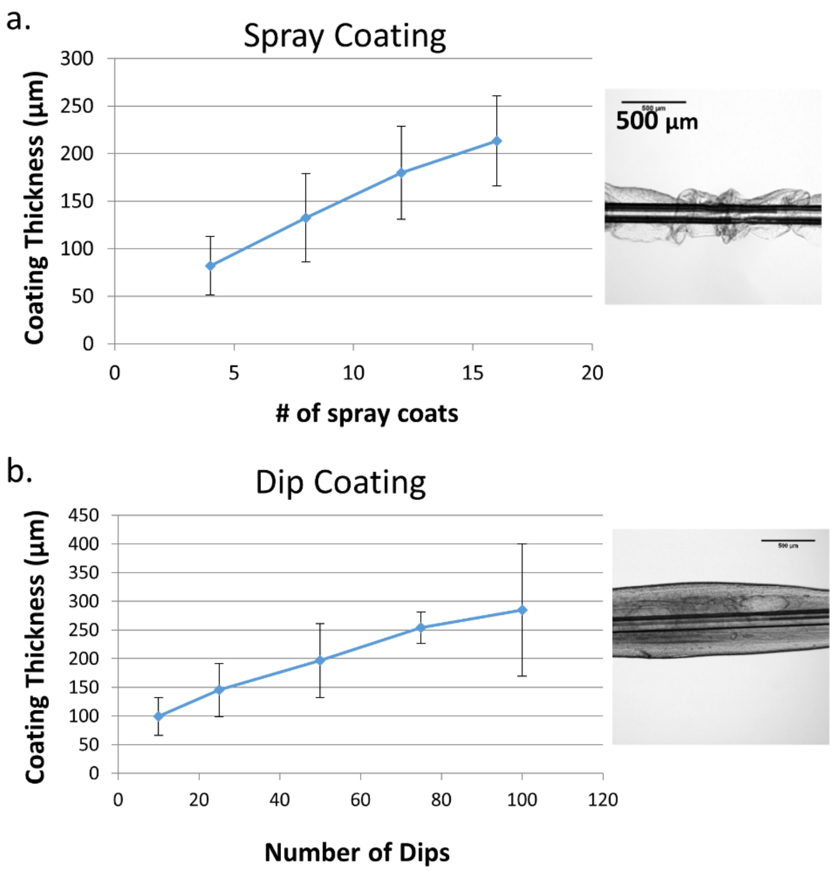

Supplementary Figure S3: PEG-DMA coatings may also be formed on the surface of borosilicate probes by spray coating and dip coating. **(A)** Coating thickness may be controlled by the number of spray coats. Spray coating involves dissolving the PEG-DMA molecules with photoinitiator in dichloromethane (10% w/v), and coating the solution on the surface of the probe via pressure driven flow. UV light exposure crosslinks the polymer network following each coating procedure. An example picture of the coating formed via spray coating is shown to the right. **(B)** The coating may also be formed via a dip coating process. The borosilicate capillaries were repeatedly dipped into an aqueous PEG-DMA solution, and exposed to UV light after removal. The total coating thickness may be controlled by the number of dip coating replicates. A representative image of the coating formed by dip coating is shown to the right of the graph. Scale bar – 500 µm.
